# Endometriosis of the Vermiform Appendix Presenting as Acute Appendicitis

**DOI:** 10.7759/cureus.5816

**Published:** 2019-10-01

**Authors:** Adeolu Adeboye, Gabriel O Ologun, Daniel Njoku, Jean Miner

**Affiliations:** 1 General Surgery, Guthrie Clinic/Robert Packer Hospital, Sayre, USA; 2 General Surgery, Robert Packer Hospital/Guthrie Clinic, Sayre, USA; 3 Biological Science, Howard University, Washington, DC, USA; 4 General Surgery, Robert Packer Hospital/ Guthrie Clinic, Sayre, USA

**Keywords:** appendix, appendicitis, endometriosis, abdominal pain, appendectomy

## Abstract

Endometriosis is characterized by the growth of endometrial tissue outside the uterine cavity. Endometriosis of the appendix is rare and its preoperative diagnosis is difficult. We report the case of a postmenopausal woman who presented with right lower quadrant abdominal pain concerning for acute appendicitis. Histopathological examination of her appendix revealed endometriosis and her abdominal pain resolved after appendectomy.

## Introduction

Endometriosis is the presence of functioning ectopic endometrial tissues outside the lining of the uterine cavity. It is usually asymptomatic; when symptoms are present, they are often based on the location of the implants [1]. Endometriosis of the appendix is rare. Patients may present with a wide range of symptoms, including acute appendicitis, intestinal perforation, intestinal obstruction or lower gastrointestinal bleeding [1-3]. Right lower quadrant abdominal pain has been described as one of the most common symptoms of appendiceal endometriosis [1]. Preoperative diagnosis is difficult, as imaging findings are usually indistinguishable from acute appendicitis. The definitive diagnosis is usually established by histopathological examination of the appendix. We report a case of endometriosis of the appendix presenting as right lower quadrant abdominal pain in a 50-year-old woman. To our knowledge, there have been no previous reports of isolated appendiceal endometriosis in a postmenopausal female without pelvic spread as found in this case.

## Case presentation

A 50-year-old postmenopausal female who presented to the emergency department with a three-week history of sharp, non-radiating, intermittent, moderate right lower quadrant abdominal pain. Her history is significant for hypertension, adenomyosis with abnormal uterine bleed for which she underwent total abdominal hysterectomy with bilateral salpingectomy. She is a non-smoker.

**Figure 1:**
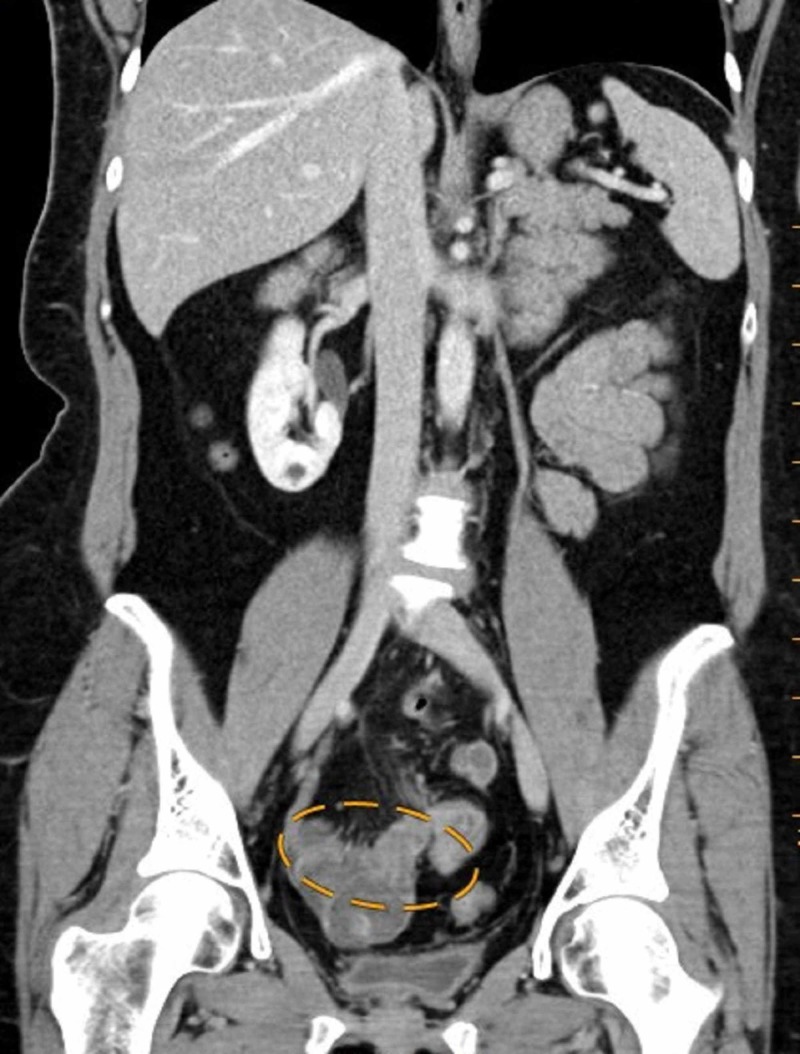
Coronal computed tomography of the abdomen and pelvis revealing a thick, irregular appendix periappendiceal stranding concerning for acute appendicitis (yellow oval ring).

**Figure 2:**
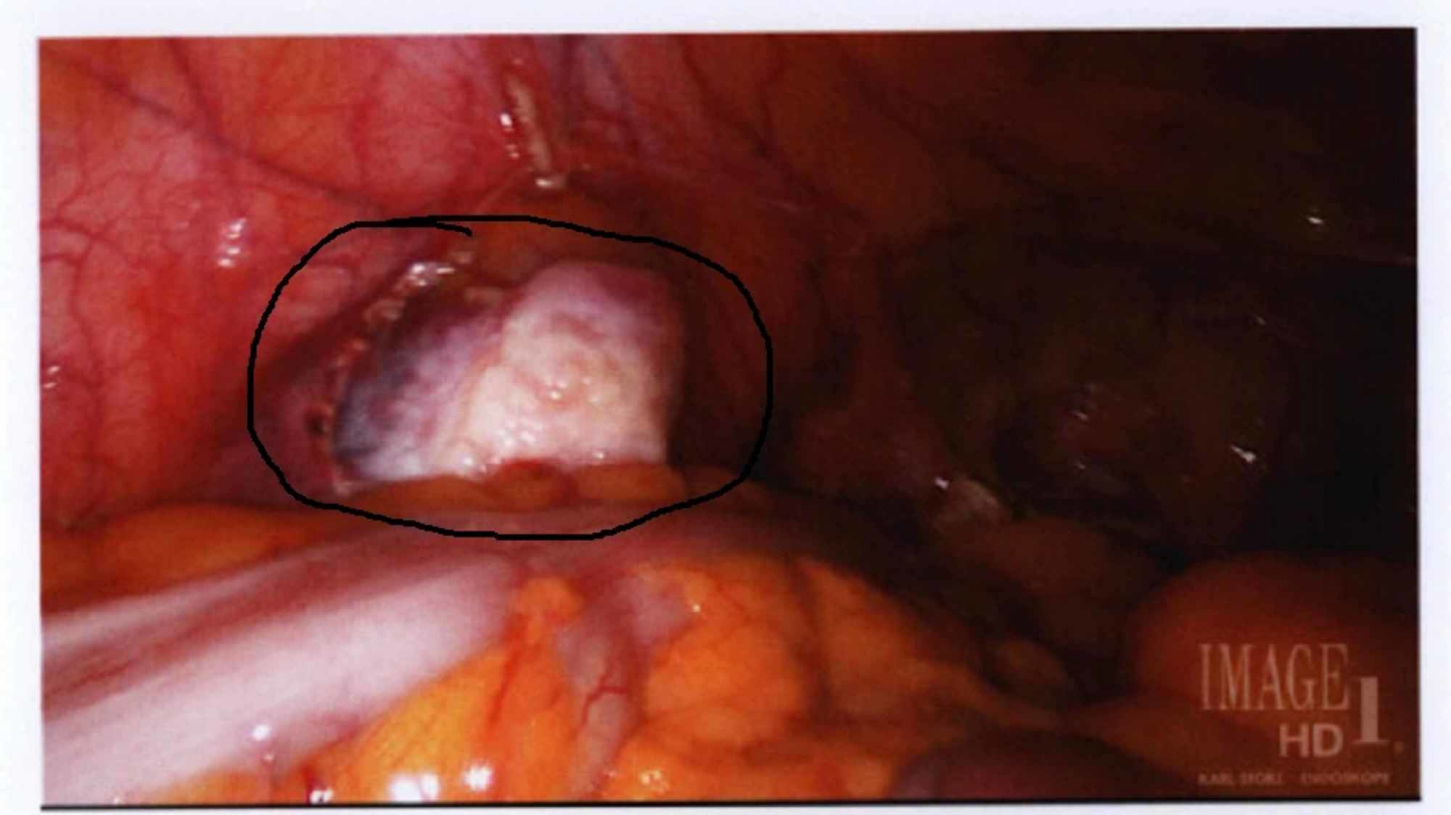
Intraoperative imaging of pelvic nodule noted in the tip of the appendix (black oval ring).

On examination, she was afebrile and hemodynamically normal. Laboratory evaluation revealed white blood cell count 11.6 K/uL, hemoglobin 12.1 g/dL, platelet count 262 K/uL. Computed tomography (CT) scan of the abdomen and pelvis with contrast showed a very thickened, irregular appendix with trace adjacent fluid that was concerning for appendicitis; the thickness of the appendix also raised some concern for potential underlying appendiceal neoplasm (Figure 1). The patient underwent colonoscopic evaluation, which was unremarkable. Surgery was then performed under general anesthesia. Intraoperatively, a 1.5-cm nodule was noted with the tip of the appendix (Figure 2), there was no evidence of pelvic endometriosis. An appendectomy was performed and the specimen was sent off for pathology evaluation.

Histopathology demonstrated endometriosis involving the appendix without involvement of other pelvic organs. The patient had an uneventful recovery and resolution of her abdominal discomfort.

## Discussion

Endometriosis is characterized by the growth of endometrial tissue outside the uterine cavity. It is well known as a cause of pelvic pain and infertility, affecting approximately 70% of women with chronic pelvic pain, and up to 50% of women with infertility. However, it can also cause acute pelvic or abdominal pain that is severe enough to prompt the patient to seek emergency medical care [1]. The majority of endometrial implants occur in the dependent parts of the female pelvis. A study that assessed the anatomic distribution of ectopic endometrium by investigating the location of implants and adhesions in 182 patients found that the most common sites of implants are the ovaries (54.9%). This was followed by the posterior broad ligament (35.2%), anterior cul-de-sac (34.6%), the posterior cul-de-sac (34.0%), and the uterosacral ligament (28.0%). Another study that looked at 1573 consecutive patients with endometriosis reports involvement of the GI tract in 5.4% of the patients. Other studies have shown that when endometriosis involves the GI tract, it commonly involves the recto-sigmoid (72.4%), the recto-vaginal septum (13.5%), small intestine (7.0%), cecum (3.6%) and the appendix (3%) [1]. Endometriosis of the GI tract is therefore uncommon, and when it occurs, it rarely involves the appendix. Involvement of the appendix may present as appendicitis or appendicular mass that may mimic a neoplasm [1].

The prevalence of appendiceal endometriosis is about 0.4 to 1% in the general population and about 4 to 22% among patients with endometriosis [4]. Endometriosis is a risk factor for developing appendiceal endometriosis; patients with deep infiltrating endometriosis have a six-fold higher risk of developing appendiceal endometriosis compared with women without endometriosis [4].

Clinically patients may present with acute appendicitis, or manifest atypical symptoms such as abdominal colic, nausea, and melena and in some situations, inverted or bulbous appendiceal orifice on colonoscopy, or sometimes the patient may be asymptomatic [5,6]. Laboratory and imaging studies are usually non-specific in the diagnosis of appendiceal endometriosis. The diagnosis can be made by histopathology, when endometrial glands and stroma are present outside of the uterus. It usually involves the serosa and subserosa in the intestine [5,6]. CT scan may demonstrate evidence of acute appendicitis or appendiceal abnormality [6].

The treatment of appendiceal endometriosis is primarily surgical. Medical treatments such as hormonal therapy are secondary [5]. The goal of surgical treatment is to remove the disease and restore the bowel continuity and function. This may require appendectomy, ileocecectomy or right hemicolectomy in the case of endometriosis of the appendix. In patients with severe endometriosis incidental appendectomy is recommended because endometriosis of the appendix may be missed on visual inspection [6].

Several theories exist regarding the pathogenesis of extrauterine endometriosis including: implantation or retrograde menstruation theory; direct transplantation and dissemination theory; coelomic metaplasia theory; the induction theory; the embryonic rest theory and the cellular immunity theory [5]. The coelomic metaplasia theory that the peritoneal cavity contains progenitor cells capable of differentiating into endometrial tissue, the induction theory proposes that sloughed endometrium produces substances that cause endometriosis. On the other hand, the embryonic rest theory hypothesizes that specific stimulus to a Mullerian origin cell nest produces endometriosis, and the cellular immunity theory suggests that alterations in cell-mediated and humoral immunity allow ectopic endometrial cells to proliferate [5].

In our patient, her history of adenomyosis further increased her risk for appendiceal endometriosis; adenomyosis has been reported to have a high incidence of association with uterine abnormalities. Endometriosis and adenomyosis have been described as variants of the same disease process, with both resulting from the exaggeration of the same physiologic mechanism [1].

## Conclusions

Endometriosis of the appendix is rare and difficult to diagnose perioperatively as these patients present with a range of symptoms including symptoms of acute appendicitis as in our case report. High index of suspicion is needed for accurate diagnosis in women with a history of gynecologic disease and should be included in the differential diagnosis for right lower quadrant pain. The mechanism of endometriosis is still unclear.
